# Simulation-based assessment of upper abdominal ultrasound skills

**DOI:** 10.1186/s12909-023-05018-1

**Published:** 2024-01-03

**Authors:** Kristina E. Teslak, Julie H. Post, Martin G. Tolsgaard, Sten Rasmussen, Mathias M. Purup, Mikkel L. Friis

**Affiliations:** 1 NordSim, Center for Skills Training and Simulation, Aalborg University Hospital, Aalborg, Denmark; 2 Copenhagen Academy for Medical Education and Simulation, Rigshospitalet, Copenhagen, Denmark; 3 Department of Clinical Medicine, Aalborg University, Aalborg, Denmark; 4 Department of Radiology, Aalborg University Hospital, Aalborg, Denmark

**Keywords:** Simulation-based ultrasound training, Validity evidence, Ultrasound assessment

## Abstract

**Background:**

Ultrasound is a safe and effective diagnostic tool used within several specialties. However, the quality of ultrasound scans relies on sufficiently skilled clinician operators. The aim of this study was to explore the validity of automated assessments of upper abdominal ultrasound skills using an ultrasound simulator.

**Methods:**

Twenty five novices and five experts were recruited, all of whom completed an assessment program for the evaluation of upper abdominal ultrasound skills on a virtual reality simulator. The program included five modules that assessed different organ systems using automated simulator metrics. We used Messick’s framework to explore the validity evidence of these simulator metrics to determine the contents of a final simulator test. We used the contrasting groups method to establish a pass/fail level for the final simulator test.

**Results:**

Thirty seven out of 60 metrics were able to discriminate between novices and experts (*p* < 0.05). The median simulator score of the final simulator test including the metrics with validity evidence was 26.68% (range: 8.1–40.5%) for novices and 85.1% (range: 56.8–91.9%) for experts. The internal structure was assessed by Cronbach alpha (0.93) and intraclass correlation coefficient (0.89). The pass/fail level was determined to be 50.9%. This pass/fail criterion found no passing novices or failing experts.

**Conclusions:**

This study collected validity evidence for simulation-based assessment of upper abdominal ultrasound examinations, which is the first step toward competency-based training. Future studies may examine how competency-based training in the simulated setting translates into improvements in clinical performances.

**Supplementary Information:**

The online version contains supplementary material available at 10.1186/s12909-023-05018-1.

## Background

Ultrasound has been used for more than half a century and is used within several specialties such as radiology, cardiology, obstetrics-gynecology, and emergency medicine among others [1].

Although ultrasound is considered safe, it is highly operator-dependent with the quality of the scan entirely depending on the competencies of the operator [2, 3]. The problem is that ultrasound learning curves are often long, albeit with large individual differences in the speed at which the necessary competencies are attained. Current recommendations from international ultrasound societies state that new trainees must complete a certain volume of scans [4]. However, a traditional focus on time- and volume-based curricula may be insufficient for some trainees and unnecessary long for others. Current best practices within the field of medical education involve the use of mastery-learning [5]. Mastery-learning involves continued assessment of trainees’ skill progression until they demonstrate a predefined competence level. The drawback of this approach is that mastery-learning is highly resource-intensive when conducted in the clinical setting, where a senior clinician must supervise and assess the trainee for extended periods of time [5].

This challenge has to some level been resolved by the introduction of simulation-based ultrasound training as a part of basic training. Simulation-based ultrasound training enables automated assessments and feedback from the simulator until the trainee has attained the elementary skills needed to commence supervised clinical training [6, 7].

The use of ultrasound simulators allows learning in a safe, controlled, and stress-free environment. Most commercially available simulators provide standardized training modules with automated assessments (metrics) to provide feedback during training [8]. However, previous studies have indicated that more than half of these feedback metrics fail to discriminate between complete novices and ultrasound experts [9, 10]. Consequently, the validity of simulation-based assessments must be critically evaluated before being used for training and assessment purposes. In the context of our study, we aimed to explore the validity of simulation-based assessments of upper abdominal ultrasound examinations, which are frequently performed by radiologists as well as emergency physicians and surgeons as point-of-care examinations. Being one of the most common types of ultrasound examinations and thereby also representing an area with massive training needs, it is important to determine how to use simulation-based assessment in a valid and reliable way to enable mastery-learning with optimal use of clinician expert time. The aim of this study was to explore the validity and reliability of simulation-based assessments of ultrasound examinations of the upper abdomen. This is important because having good assessments of competence is the basis for mastery-learning, which is currently considered as the preferred standard for simulation-based medical education before learners enter supervised clinical practice.

## Methods

### Setting/design

This study was conducted from April 2021 to June 2021 at NordSim and the department of Radiology, Aalborg University Hospital in Denmark. The study investigated the validity evidence of automatic feedback metrics using abdominal ultrasound modules and cases on a commercially available ultrasound simulator (Scantrainer, Medaphor). We used Messick’s framework for validity testing according to existing standards for educational testing, which includes five different sources of validity [11, 12]: content evidence, response process, relations to other variables, internal structure, and consequences. The study was approved by Danish Data Protection (Protocol no. 2021–106) and exempt from The Ethical Committee of Region North Jutland (File No. 2021–000438).

### Participant recruitment

The participants consisted of five experts and 25 novices. The expert group included radiologists from Aalborg University Hospital and the novices included third year medical students from Aalborg University. The inclusion criteria for the novices were that they had completed their course in basic abdominal anatomy and that they were able to participate within the designated time frame. The exclusion criteria were previous experience with ultrasound in the clinical setting or with ultrasound simulation. All participants in the study received written informed consent before beginning the study. Demographics of the participants are shown in Table 1.

**Table 1:** Baseline demographics of participants

Characteristics	Novices (*n* = 25)	Experts (*n* = 5)
Median, age (range)	23 (21–28)	40 (38–52)
Women, n	12	2
Men, n	13	3
Median years of experience (range)	0	10 (4.5–18)

### Simulator equipment

A virtual reality transabdominal ultrasound simulator (ScanTrainer, MedaPhor) was used for all assessments. The simulator consists of two monitors and a robotic arm with an armrest. The robotic arm simulates the ultrasound probe and provides haptic feedback during scanning. The right monitor is placed above the haptic device illustrating a 3D figure of a virtual patient, anatomical structures and the movement of the probe on the virtual patient. The left monitor illustrates an ultrasound image including options for image optimization [6].

The simulator includes a number of modules with exercises relating to the examination of the upper abdomen. Examples of tasks are’Label the gallbladder’,’Sweep through the long axis of the gallbladder’,’Measure the AP diameter of the gallbladder wall’ and’Demonstrate a view of the long axis of the spleen including the left kidney’. After completion of a module, multiple metrics (i.e., automated assessments provided by the simulator) are provided as feedback in the format of a pass/fail decision for each metric.

### Procedures

A simulator instructor provided all participants a standardized introduction regarding the use of the simulator involving information about the setup of the study and its purpose. The introduction included information about manipulation of the probe to visualize different planes and image optimization regarding gain, zoom, depth, and focus. We used a protocol for instructing participants that ensured standardization across the different participants. Afterwards, the participants performed a warm-up session to become familiar with the simulator. They had to perform the same program twice with a 5-min break between each round. In this break, it was ensured that no data was missing. To ensure standardization and prevent possible errors, the simulator instructor did the technical work and ensured the tasks were understood. In case of technical errors, the task had to be redone. An upper time limit was set to 70 min to complete one round resulting in two minutes per task. All participants were required to complete the entire test twice to allow evaluation of test/retest reliability. The testing effect from the first test to the second test was minimized by blinding the participants from any simulator feedback during testing. The simulator instructor’s presence was necessary to observe any threats to response process validity (see below).

### Evaluation of test validity

We used Messick’s framework [12] to evaluate validity of the simulation-based assessments according to existing recommendations by the latest Standards for Educational and Psychological Testing [11]. This involved a five-step process that included evaluation of the following sources of validity evidence:

- Step 1: Content evidence

To map the content of the test program for existing best practices, a senior registrar in radiology set up a test program reflecting an upper abdominal ultrasound examination as performed in the context of a diagnostic radiological assessment. According to EFSUMB, the guidelines for ultrasound of the upper abdomen is examination of the liver, gallbladder, bile ducts, pancreas, spleen, kidneys, and adrenal glands [4]. When designing the test, we aimed to include as many unique cases as possible, to represent all content matter areas reflecting the EFSUMB guidelines, and to avoid redundant tasks to keep the test duration below one hour to improve feasibility.

- Step 2: Response process

The response process was examined through a range of evaluations, including how well the construct of the test (that is, competence in performing upper abdominal ultrasound) matched how the participants actually interacted with the test, how they perceived the test format, and how well the assessments captured the participants’ performances.

- Step 3: Relations to other variables

Relations to other variables were determined based on how well the test scores were able to discriminate between participants with different levels of ultrasound competence. The automated simulator metrics provided a score of 1 (passed) or 0 (failed) for each metric. For each metric, we examined their discriminatory capability (did they discriminate between novices and experts) using Fisher’s exact test. By including only the metrics that were able to discriminate, we constructed a final test and explored distribution of test scores between the two groups of participants.

- Step 4: Internal structure

The internal structure was assessed using Cronbach’s alpha and test/retest reliability of the metrics constituting the final test was evaluated using Intraclass Correlation Coefficients (two-way, absolute consistency).

- Step 5: Consequences

Based on simulator metrics with validity evidence, two performance levels were defined to determine the consequences of a certain test score. The first performance level was the pass/fail level which was determined by the contrasting groups method [13]. This was done by identifying the intersection between the distribution of standardized scores between the novices and experts to allow as few false-negatives (failing experts) and false-positives (passing novices) as possible [14]. The second performance level was the mastery level, which was determined as the median sum score of the experts.

### Statistical analyses

Sample size was determined using G*Power version 3.1.9.6. A total size of 30 participants was needed to detect differences corresponding similar validation studies (an effect size of at least 1.2 SD) when using an alpha level of 0.05 and a power of 0.80 We chose a skewed distribution of participants to allow more novices than experts to participate because previous studies have shown that novices demonstrate greater variation in performances compared to experts, who most often perform very consistently as a group [9, 10, 15].

Statistical analyses were conducted using SPSS version 27.0. To determine whether the simulator metrics discriminated between the novices and experts, Fisher’s exact test was performed. This was done by assigning the passed metrics with the value 1 and the failed metrics with the value 0.

For the metrics with validity evidence a sum score of the simulator test was calculated as the percent of maximum score. These sum scores of novices and experts were compared by the Mann–Whitney U test. Cronbach α was used to assess the internal consistency of the simulator test. A level above 0.70 was considered acceptable [16]. The intraclass correlation coefficient was calculated for assessment of the test/retest reliability of the two rounds in the simulator test. A level above 0.50 was considered acceptable [8].

## Results

A total of five experts and 25 novices completed the study as seen in Table 1.

### Step 1: content evidence

A test program containing five modules and 60 metrics was constructed to reflect the EFSUMB guidelines on ultrasound of the upper abdomen. The simulator did not afford tasks to reflect examination of the bile ducts and adrenal glands.

### Step 2: response process

Comments from the expert participants were collected during the assessments. One expert had difficulties with imagining scanning a patient without a phantom. Another expert pointed out the fact that the ultrasound image did not show compression corresponding to pressure applied to the simulator. Finally, four out of a total of five radiologists mentioned Doppler as a missing feature when investigating aorta, renal pelvis, and splenic hilum. There we no issues in the completeness of registration and documentation of assessments as observed by the simulator instructor.

### Step 3: relations to other variables

Of the 60 metrics included for validity testing, 37 (62%) were able to discriminate between novices and experts (*p* < 0.05). The distribution of metrics across the different modules is shown in Table 2 and metrics with established validity evidence and the corresponding p-values for expert-novice comparisons are listed in Table 3. There were significant differences between scores of the final test for the novices and experts, mean 24.92% (SD 9.59) vs. mean 81.62% (SD 11.66), respectively, *p* < 0. 001.The experts used less time to complete the test program than the novices, 42 min (range 34–50) vs. 56 min (range 32–102), (*p* = 0.006).

**Table 2:** Distribution of metrics divided into categories

Metrics	Valid	Nonvalid	Total
Organ/area labelled correctly	18	1	19
Transducer orientated in correct plane/axis	8	5	13
Area correctly measured and in correct plane/angle	5	0	5
Organ correctly examined	0	6	6
Organ correctly centralised	4	6	10
Organ correctly visualised	2	5	7
Total	37	23	60

**Table 3:** All metrics with validity evidence and the associated *p*-values for expert-novice comparisons

Metrics with validity evidence	Significance
3.1.1.1 Labelling the aorta	*p* = 0.008
3.1.1.2 Transducer orientated in the sagittal plane	*p* = 0.025
3.1.2.1 Labelling the inferior vena cava	*p* = 0.000112
3.1.2.2 Transducer orientated in the sagittal plane	*p* = 0.001
3.1.3.1 Labelling the ligamentum teres	*p* = 0.005
3.1.4.1 Labelling the caudate lobe	*p* = 1.3277·10^−8^
3.1.5.1 Labelling the gallbladder	*p* = 8.754 ·10^−10^
3.1.6.1 Labelling the right hemi-diaphragm	*p* = 0.003
3.1.7.1 Labelling right the sub-pleural space	*p* = 0.021
3.1.8.1 Labelling Morison’s pouch	*p* = 1.459 ·10^−10^
4.1.2.1 Labelling the neck of the gallbladder	*p* = 0.000026
4.1.3.1 Labelling the body of the gallbladder	*p* = 0.010
4.1.4.1 Labelling the fundus of the gallbladder	*p* = 0.000157
4.1.6.1 Measure the AP diameter of the gallbladder wall	*p* = 3.5891 ·10^−8^
4.1.6.2 AP diameter of the gallbladder wall measured in the correct position	*p* = 0.005
5.2.2.1 Labelling the head of the pancreas	*p* = 0.000003
5.2.2.2 Transducer orientated in the transverse plane	*p* = 0.035
5.2.3.1 Labelling the uncinate process	*p* = 0.000103
5.2.3.2. Transducer orientated in the transverse plane	*p* = 0.039
5.2.4.1 Labelling the neck of the pancreas	*p* = 0.000431
5.2.4.2 Transducer orientated in the transverse plane	*p* = 0.039
5.2.5.1 Labelling the body of the pancreas	*p* = 1.3277 ·10^−8^
5.2.5.2 Transducer orientated in the transverse plane	*p* = 0.033
5.2.6.1 Labelling the tail of the pancreas	*p* = 0.011
5.2.6.2 Transducer orientated in the transverse plane	*p* = 0.015
5.2.9.1 Uncinate process correctly centralised	*p* = 0.001
6.1.1.4 Transducer orientated in the sagittal plane	*p* = 0.035
6.1.3.1 Labelling a column of Bertin	*p* = 0.008
8.1.2.1 Measure the length of the spleen	*p* = 1.0622 ·10 ^7^
8.1.2.2 Length of the spleen measured in the correct position	*p* = 5.8039 ·10 ^7^
8.1.2.3 Length of the spleen measured at the correct angle	*p* = 0.000189
8.1.3.1 Spleen correctly centralised	*p* = 0.025
8.1.4.1 Spleen correctly centralised	*P* = 0.05
8.1.4.3 Diaphragm visualised	*p* = 0.001
8.1.6.1 Spleen correctly centralised	*p* = 0.010
8.1.6.2 Splenic hilum visualised	*p* = 0.000015

### Step 4: internal structure

The internal consistency of the final test program was high, Cronbach alpha = 0.93. The test/retest reliability was also high, ICC = 0.89.

### Step 5: consequences

**Fig. 1:**
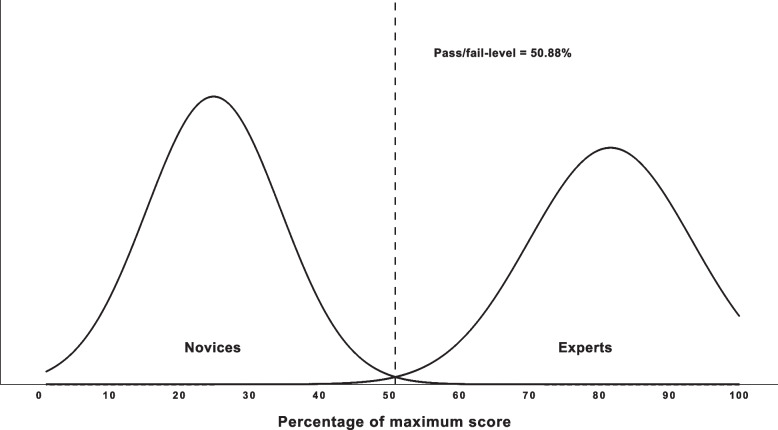
The performance level of novice and expert participants during pretest contributed to establishment of a pass/fail-level by the contrasting groups method [13]. The pass/fail-level was determined as the intersection between standardized distributions of the novice and expert groups to ensure as few competent operators failing (false-negatives) and incompetent operators passing (false-positives)

A pass/fail level using the contrasting groups method was determined to be 50.9%, which allowed no passing novices (false-positive) and no failing experts (false-negative) as illustrated in Fig. 1. The expert level was determined corresponding to the median sum score of the expert group at 85.1% (range: 56.8%-91.9%) and for novices at 25.7% (range: 8.1%-40.5%).

## Discussion

Ultrasound of the upper abdomen is one of the most common types of ultrasound examination, making it one of the top priorities in educating radiologists and physicians using point-of-care ultrasound [17]. The use of simulation-based ultrasound training has led to improved clinical performances, shorter examination time, lower patient discomfort, and less need for faculty supervision [18, 19]. However, a core requirement for the effective use of simulation for assessment and learning is evidence of test validity [20]. This study is the first step toward enabling mastery-learning, which relies on having good assessments to guide the learners on their path to the a pre-establish mastery learning level (for instance, defined by the expert performance). In this study we found that only 37 out of 60 (62%) in-built simulator metrics were able to discriminate between novices and experts when assessing upper abdominal ultrasound skills. This underlines the need for validation studies prior to the use of simulation for assessment as well as mastery learning, in which trainees are required to practice until they reach a certain predefined skill level. In our study, we established two levels that can be used for future research and training purposes. The first level was a pass-fail level that may be used as a landmark for the minimum level of skills needed to advance to the next level of training, such as clinical training. The second level was the expert level, which in previous studies has shown to be attainable by novice trainees with sufficient training and associated with better transfer of skills [21, 22].

The majority of the metrics considered to possess validity evidence involved’labelling an organ/area’, that is, pertaining to image interpretation. This may reflect lower levels of anatomical knowledge in the novice group but may also involve image recognition skills that are developed during initial practice. On the other hand, none of the metrics concerning the systematic examination of an organ or area were able to discriminate between the two groups as most novices passed these metrics suggesting a difficulty level that was too low.

In contrast to these results, a previous study investigating simulation-based assessment of FAST skills identified image optimization (organ/area correctly centralized, transducer in the correct plane/axis) followed by systematic scanning technique (organ/area correctly visualized and/or examined) as the metrics that discriminated the best between operators with different levels of expertise [15]. These differences may pertain to the type of task explored, as diagnostic ultrasound of the upper abdomen has a completely different focus and scope than a point-of-care examination aiming to detect the presence or absence of free fluid. The difference between diagnostic versus point-of-care examinations offer different prerequisites for the competent completion of each of these two types of tasks. In other words, validity evidence does not seem to transfer between tasks that on a surface level bears many similarities, underlining the need for repeated validity testing before the adoption of simulation-based assessments for practice and certification.

A strength of this study was the controlled study design and the rigorous approach to validity testing. The simulator instructor gave standardized instructions and was responsible for the technical assistance during all testing to prevent protocol deviations, cheating or misunderstandings when completing the tasks. Moreover, the participants were assessed by standardized automatic feedback metrics, supporting the reproducibility of our findings in other populations. While outside the scope of the present study, future research should explore how different sources of validity evidence change across multiple different types of assessment, including other types of technology-enhanced assessments as well as rater-based approaches.

A limitation is the rather homogeneous study population as the novices only involved medical students from a single university and the experts only involved radiologists from one radiology department. The homogeneity may lead to more consistent results but at the cost of generalizability across a more diverse group of learners with different levels of prior ultrasound knowledge and experience. Using groups that are homogenous and far apart (expert-novice comparisons) is a limitation to the validity argument. Yet, we used these comparisons to sort out metrics that failed to discriminate under the assumption that if they failed to discriminate when the differences were large, they will likely also fail to discriminate between small differences in performance.

The participants were given an upper time limit of 70 min for the pretest, which may have compromised the performance of the novices making them perform worse than expected. Furthermore, a simulator instructor sat next to the participant during all testing which may have given the participant a feeling of being monitored, although this also ensured high consistency in data collection and data integrity. The radiologists may also have been unaccustomed to the feeling of being evaluated and this could cause them to underperform compared with when performing ultrasound examinations in clinical practice (negative transfer). On the other hand, having access to the 3D illustration in the extra screen may have enabled the novices to navigate better than only having access to the 2D ultrasound image and we may thereby have overestimated their performance. Finally, a few of the radiologists stated that a number of the tasks in the test did not align with the normal workflow and focus areas during clinical ultrasound examination, which was expected given the natural gap in fidelity between ultrasound simulation and real ultrasound examinations. An added challenge is that the simulation task will never align perfectly with clinical task, in our case for instance by the absence of Doppler imaging or the inability to compress tissue with the probe in the simulated setting.

## Conclusion

This study collected validity evidence for simulation-based assessment of upper abdominal ultrasound examinations, which is the basis for competency-based training such as mastery-learning. Future studies should examine how much training is needed for novices to attain expert level performance in performing upper abdominal ultrasound as well as determine its impact on subsequent clinical performances.

### Supplementary Information

**Additional file 1.**

**Additional file 2.**

## Data Availability

The datasets used and/or analysed during the current study are available from the corresponding author on reasonable request. Raw data regarding baseline demographics and metric scores can be found in supplementary material.
